# Enhanced L-β-Aminoisobutyric Acid Is Involved in the Pathophysiology of Effectiveness for Treatment-Resistant Schizophrenia and Adverse Reactions of Clozapine

**DOI:** 10.3390/biom13050862

**Published:** 2023-05-19

**Authors:** Kouji Fukuyama, Eishi Motomura, Motohiro Okada

**Affiliations:** Department of Neuropsychiatry, Division of Neuroscience, Graduate School of Medicine, Mie University, Tsu 514-8507, Japan; k-fukuyama@clin.medic.mie-u.ac.jp (K.F.); motomura@clin.medic.mie-u.ac.jp (E.M.)

**Keywords:** clozapine, L-β-aminoisobutyric acid, astrocyte, microdialysis, schizophrenia

## Abstract

Clozapine is an effective antipsychotic for the treatment of antipsychotic-resistant schizophrenia; however, specific types of A/B adverse effects and clozapine-discontinuation syndromes are also well known. To date, both the critical mechanisms of clinical actions (effective for antipsychotic-resistant schizophrenia) and the adverse effects of clozapine remain to be elucidated. Recently, we demonstrated that clozapine increased the synthesis of L-β-aminoisobutyric acid (L-BAIBA) in the hypothalamus. L-BAIBA is an activator of the adenosine monophosphate-activated protein kinase (AMPK), glycine receptor, GABA_A_ receptor, and GABA_B_ receptor (GABA_B_-R). These targets of L-BAIBA overlap as potential targets other than the monoamine receptors of clozapine. However, the direct binding of clozapine to these aminoacidic transmitter/modulator receptors remains to be clarified. Therefore, to explore the contribution of increased L-BAIBA on the clinical action of clozapine, this study determined the effects of clozapine and L-BAIBA on tripartite synaptic transmission, including GABA_B_-R and the group-III metabotropic glutamate receptor (III-mGluR) using cultured astrocytes, as well as on the thalamocortical hyper-glutamatergic transmission induced by impaired glutamate/NMDA receptors using microdialysis. Clozapine increased astroglial L-BAIBA synthesis in time/concentration-dependent manners. Increased L-BAIBA synthesis was observed until 3 days after clozapine discontinuation. Clozapine did not directly bind III-mGluR or GABA_B_-R, whereas L-BAIBA activated these receptors in the astrocytes. Local administration of MK801 into the reticular thalamic nucleus (RTN) increased L-glutamate release in the medial frontal cortex (mPFC) (MK801-evoked L-glutamate release). Local administration of L-BAIBA into the mPFC suppressed MK801-evoked L-glutamate release. These actions of L-BAIBA were inhibited by antagonists of III-mGluR and GABA_B_-R, similar to clozapine. These in vitro and in vivo analyses suggest that increased frontal L-BAIBA signaling likely plays an important role in the pharmacological actions of clozapine, such as improving the effectiveness of treating treatment-resistant schizophrenia and several clozapine discontinuation syndromes via the activation of III-mGluR and GABA_B_-R in the mPFC.

## 1. Introduction

Clozapine is well established as one of the most effective antipsychotic agents and effective in the treatment of antipsychotic-resistant schizophrenia, as demonstrated by various meta-analyses [1,2,3,4,5]; however, clozapine is also known to have numerous specific/serious adverse effects, such as type B reactions (myocarditis, cardiomyopathy, agranulocytosis, and convulsions) and type A reactions (weight increase and metabolic disturbance) [6,7,8]. Therefore, psychiatrists must occasionally promptly discontinue clozapine prescription or switch to other antipsychotics due to these serious type B adverse effects. However, prompt discontinuation often leads to clozapine-discontinuation symptoms, including clozapine-discontinuation-induced worsening of psychosis and catatonia [9,10,11]. These distinct clinical advantages and disadvantages of clozapine compared to other atypical antipsychotics suggest that clozapine likely has different mechanisms of action compared to other antipsychotics.

Traditionally, dopaminergic and/or glutamatergic dysfunctions play an important role in the pathophysiology of schizophrenia, with several atypical antipsychotics improving mesolimbic dopaminergic hyperfunction and mesocortical hypofunction [12]. An impaired N-methyl-D-aspartate type glutamate receptor (NMDA-R) has also been considered to contribute to the pathophysiology of schizophrenia [13]. Considering these clinical and preclinical findings, within the current pathophysiology of schizophrenia, it was hypothesized that the pathomechanisms of schizophrenia are composed of heterogeneous etiological factors, rather than caused by a single etiological factor, via complex interactions in networks of interacting pathogenic influences [13,14,15,16]. In the pathophysiology of schizophrenia, numerous preclinical studies reported the conspicuous pharmacodynamic profiles of clozapine compared to other antipsychotics. Indeed, clozapine inhibits various monoamine receptors, but its D2 receptor inhibition is seemingly weaker than that of other antipsychotics [11], whereas clozapine enhances NMDA-R by increasing the release of both glutamate and D-serine through astroglial exocytosis and non-exocytosis processes [17,18,19,20]. These enhancements in glutamatergic transmission induced by clozapine may contribute to the pro-cognitive actions of clozapine [11,17,18,19]. In other words, these findings strongly indicate that tripartite synaptic transmission possibly plays fundamental roles in clozapine’s mechanisms of clinical action [11,21,22].

In mechanistically analyzing the pathophysiology of weight increase and metabolic disturbance of antipsychotics, we recently demonstrated that the chronic administration of clozapine enhanced the synthesis of L-β-aminoisobutyric acid (L-BAIBA), known as a protective myokine, in the hypothalamus [23,24]. BAIBA enantiomer, a structural GABA isomer, was originally discovered in human urine in 1951 [25]; however, the physiological function of this isomer remained to be elucidated. In the peripheral organs, the BAIBA enantiomer was re-discovered as a protective myokine that regulates adipose tissue browning, enhances sensitivity to insulin, and improves obesity induced by high-fat diets [26,27,28]. Additionally, the BAIBA enantiomer increases the signaling of protein kinase B (Akt), AMPK, and the insulin receptor substrate as well as decreases the expression of gluconeogenic enzymes [28]. The activation of AMPK signaling is considered one of the major established therapeutic targets for the treatment of insulin-resistant diabetes [29,30]. Indeed, several meta-analyses reported that antipsychotic-induced weight increase and metabolic disturbances were meaningfully suppressed by metformin (AMPK activator) [31,32,33,34,35]. AMPK protectively regulates metabolism in the peripheral organs, whereas AMPK in the hypothalamus is a fundamental player in regulating both sides of the energy balance equation (feeding and energy expenditures) in the body [29]. In other words, enhancement of hypothalamic AMPK signaling is considered to contribute to the pathophysiology of antipsychotic-induced weight increase and metabolic disturbances [21,23,36,37]. Indeed, a number of pharmacodynamic studies revealed that high-risk antipsychotics for weight increase and metabolic disturbances, such as clozapine, olanzapine, quetiapine, and zotepine, activate AMPK signaling, but lower-risk antipsychotics such as lurasidone and brexpiprazole decrease or do not affect AMPK signaling in the central nervous system (CNS) [21,23,37,38,39,40,41].

Based on the receptor binding profiles, it was clinically speculated that the major mechanism of AMPK signaling enhancement by high-risk antipsychotics for weight increase and metabolic disturbances is mediated by their high-affinity antagonism against histamine H1 and serotonin 5-HT2A receptors [21,24,37,42,43,44]. Briefly, the activation of the H1 and 5-HT2A receptors increases inositol trisphosphate (IP3) production. Increasing intracellular IP3 stimulates the calcium-induced calcium-releasing system (CICR), resulting in the activation of various intracellular signal transductions, such as adenosine triphosphate (ATP) synthase [21,23,36,37,45,46,47]. Therefore, decreased IP3 levels due to the inhibition of histamine H1 and serotonin 5-HT2A receptors suppress ATP synthesis, leading to the activation of hypothalamic AMPK signaling [21,23,36,37]. However, the chronic administration of clozapine was found to activate AMPK signaling without suppressing ATP synthesis in the hypothalamus [23,24], thereby demonstrating that clozapine increases the synthesis of L-BAIBA, which directly enhances AMPK signaling [23,24], which could inform a more rational pathophysiological hypothesis for antipsychotic-induced weight increase and metabolic disturbances. An increase in intracellular L-BAIBA under chronic clozapine administration influenced the activation of L-BAIBA release through the activated astroglial hemichannel but did not affect astroglial exocytosis [23]. Preclinical findings increasingly suggest that the pathophysiology of clozapine is likely attributable to not only neurotransmission but also tripartite synaptic transmission. Therefore, considering these tripartite synaptic transmission hypotheses of clozapine, the potentiation of L-BAIBA induced by clozapine possibly represents an attractive new target for interpreting the mechanisms of clozapine on treatment-resistant schizophrenia.

Notably, several functions of the BAIBA enantiomer have been identified in the CNS, such as activation of glycine receptors, GABA_A_ receptors, and GABA_B_ (GABA_B_-R) receptors [23,48,49]. Interestingly, a recent study on the X-ray crystal structure of GABA_B_-R along with molecular docking calculations strongly indicated the possibility that clozapine binds to GABA_B_-R, similar to baclofen [50]. This hypothesis of clozapine binding to GABA_B_-R is attractive when it comes to understanding the mechanisms underlying the clinical action of clozapine against treatment-resistant schizophrenia along with the pathophysiology of clozapine-discontinuation catatonia [51]. Although both clinical and preclinical studies suggested that clozapine enhances GABA_B_-R functions, there is no supporting experimental evidence that clozapine directly binds to GABA_B_-R; conversely, the binding of clozapine to GABA_B_-R has been rejected [52,53,54]. Considering these previous findings, the enhancement of GABA_B_-R functions by clozapine is likely mediated by indirect mechanisms rather than the direct agonistic action of clozapine. Therefore, the increasing synthesis of L-BAIBA by clozapine could support the mechanisms of the clinically observed effectiveness of clozapine in the treatment of treatment-resistant schizophrenia and clozapine-discontinuation catatonia, possibly mediated by GABA_B_-R’s functions [51].

The glutamatergic hypothesis for the pathophysiology of schizophrenia still has aspects to be clarified. The systemic administration of non-competitive NMDA-R antagonists was found to increase glutamatergic transmission in the prefrontal cortex [55]. The increasing prefrontal glutamate release by NMDA-R antagonists was generated by GABAergic disinhibition in the reticular thalamic nucleus (RTN), since the local administration of these agents into the thalamus and prefrontal cortex increased but could not increase glutamate release in the prefrontal cortex [56,57]. Conversely, local administration of clozapine into the prefrontal cortex weakly increased glutamate release but drastically suppressed the glutamate release induced by the systemic administration of NMDA-R antagonists [18]. These inhibitory effects of clozapine on frontal glutamate release induced by NMDA-R blocking are, at least partially, modulated by the activation of the group III metabolic glutamate receptor (III-mGluR) [18]. However, similar to GABA_B_-R, the binding of clozapine to III-mGluR has not yet been clarified. Based on these discrepancies between the receptor binding and signal transmission of clozapine, the present study explored whether increasing L-BAIBA synthesis with clozapine may contribute not only to its adverse effects, such as weight increase and metabolic disturbances, but also to the mechanism of efficacy underlying treatment-resistant schizophrenia.

## 2. Materials and Methods

### 2.1. Chemical Agents

Clozapine, L-β-aminoisobutyric acid (L-BAIBA), MK801 (non-competitive NMDA-R antagonist), CGP52432 (GABA_B_-R antagonist), α-cyclopropyl-4-phosphono-phenylglycine (CPPG: III-mGluR antagonist), TAT-Gap19 (connexin43 inhibitor), and tetanus toxin (TeNT: synaptobrevin inhibitor) were obtained from Funakoshi (Tokyo, Japan). All agents were prepared on the day of the experiment. Initially, clozapine was dissolved in 1 N HCl (50 mM) [17]. CGP52432, CPPG, TAT-Gap19, TeNT, and L-BAIBA were directly dissolved in experimental solutions [18,23,58].

### 2.2. Experimental Animals

Experimental animal care and experimental procedures were performed according to the ARRIVE (the Reporting of In Vivo Experiments) guidelines [59] and the ethical guidelines established by the Institutional Animal Care and Use Committee at Mie University, Japan (No.2019-3, 24 May 2019). Neonatal (0~48 h of age: n = 18) and male (6–7 weeks of age: n = 54) Sprague–Dawley rats (SLC, Shizuoka, Japan) were used for in vitro cultured astrocyte and in vivo microdialysis analyses, respectively. Adult rats were individually housed in cages with air-conditioned rooms (22 ± 2 °C) under 12 h light and 12 h dark cycles and given free access to food and water.

### 2.3. Primary Cultured Astrocytes

The primary cortical astrocyte cultures were prepared according to previous reports [17,60]. The cerebral hemispheres were removed under a dissecting microscope. The brain was chopped into fine pieces using scissors and triturated with a micropipette. The suspension was filtered through a 70 µm nylon mesh (BD, Franklin Lakes, NJ, USA) and then centrifuged. The pellets were suspended in Dulbecco’s modified Eagle’s medium (D6546; Sigma-Aldrich, St. Louis, MO, USA) containing 10% fetal calf serum (fDMEM). After 14 d of culturing (DIV14) to DIV28, the astrocytes were tripsynized and seeded directly on a translucent polyethylene terephthalate membrane (1.0 μm) with 24-well plates (BD, Franklin Lakes, NJ, USA) at a density of 100 cells/cm^2^ for the experiments. fDMEM was changed twice a week between DIV14 and DIV28. At DIV28, astrocytes were washed out repeatedly three times using artificial cerebrospinal fluid (ACSF) (0.15 M Na^+^, 0.03 M K^+^, 0.014 M Ca^2+^, and 0.008 M Mg^2+^ and 0.055 M glucose adjusted to pH = 7.3 using 0.02 M HEPES buffer) for the experiments.

#### 2.3.1. Effects of L-BAIBA on GABA_B_-R and III-mGluR

To study the acute effects of L-BAIBA on GABA_B_-R and III-mGluR, the level-dependent effects of L-BAIBA on the accumulation of cyclic adenosine monophosphate (cAMP), which is the second messenger of both GABA_B_-R and III-mGluR, were determined. After the cleaning using ACSF, cultured astrocytes were exposed to ACSF for 30 min (pre-incubation). After pre-incubation, the astrocytes were exposed to ACSF containing 10 μM forskolin and 1 mM IBMX with L-BAIBA (control) for 20 min. To study the distinct effects of L-BAIBA on GABA_B_-R and III-mGluR, the astrocytes were exposed to the same medium of control with the addition of CGP52432 (50 μM) or CPPG (100 μM) for 20 min [61,62]. Afterwards, the astrocytes were homogenized using an ultrasonic cell disrupter (VP-050N, Taitec, Koshigaya, Japan) in chilled 4 N perchloric acid with 4.3 mM EDTA [63]. The mixture was centrifuged at 10,000 × *g* for 20 min at 4 °C. Filtered aliquots (5 μL) were injected into ultra-high-pressure liquid chromatography (UHPLC) equipped with mass spectrometry (UHPLC-MS).

#### 2.3.2. Determination of Intracellular L-BAIBA Levels in the Astrocytes

The chronic exposure to a therapeutically relevant concentration of clozapine increased intracellular L-BAIBA levels in the astrocytes [23]. However, the effects of acute exposure to clozapine on intracellular L-BAIBA levels were not clarified. Furthermore, the critical time period for a clozapine-induced increase in L-BAIBA synthesis, which can contribute to acute effects on GABA_B_-R and III-mGluR, were also not clarified. Therefore, the time-dependent and concentration-dependent effects of clozapine on L-BAIBA synthesis in astrocytes were determined.

At DIV28, after washout and pre-incubation, astrocytes were incubated in ACSF containing L-BAIBA for 60~240 min. After incubation, the astrocytes were homogenized with an ultrasonic cell disrupter (VP-050N) in chilled 4 N perchloric acid with 4.3 mM EDTA [63]. The mixture was centrifuged at 10,000× *g* for 20 min at 4 °C. Filtered aliquots (5 μL) were injected into a UHPLC equipped with a fluorescence detector.

#### 2.3.3. Acute Effects of Clozapine on Astroglial Release of L-BAIBA Evoked by Ripple-Burst Stimulation

Under physiological condition, astroglial hemichannels are not functional due to their low opening probabilities, whereas persistent high-frequency electrical depolarization enhances the opening probability of astroglial hemichannels [38,40,64,65]. Therefore, to study the functions of activated hemichannels under physiological conditions, the cultured cortical astrocytes were activated via ripple-burst-evoked stimulation using a busdrive amplifier (SEG-3104MG; Miyuki Giken, Tokyo, Japan). Ripple-bursts, which were clinically observed during sleep spindle using a wide-band electrocorticogram [64], play important roles in memory integration during sleep as one of the components in neuro-cognition [66]. Ripple-burst-evoked stimulation was set to a square-wave direct-current pulse output with a magnitude of 300 mV/mm^2^ [64]. The ripple-burst-evoked stimulation was composed of 10 stimuli at 200 Hz and 10 bursts (50% duty cycle) at burst intervals of 100 ms/s [64]. The pattern of ripple-burst-evoked stimulation was regulated using the LabChart version 8.2 software (AD Instruments, Dunedin, New Zealand).

To clarify the level-dependent effects of clozapine on L-BAIBA release through activated astroglial hemichannels, astrocytes were exposed to ACSF containing clozapine for 4~20 h. Clinically, therapeutically relevant serum concentrations of clozapine were estimated to be approximately 0.3~3 μM [67,68]. Based on previous clinical findings, in the present study, cultured astrocytes were acutely administrated 0.1~100 μM clozapine for 4~20 h. To clarify the effects of clozapine on ripple-evoked L-BAIBA release through activated hemichannels, the interactions between clozapine and 20 μM TAT-Gap19 on ripple-evoked L-BAIBA release were determined. To clarify the effects of clozapine on exocytotic ripple-evoked L-BAIBA release, at DIV27, astrocytes were incubated in fDMEM containing 3 μg/mL TeNT for 24 h. After TeNT exposure, astrocytes were exposed to ACSF containing clozapine.

### 2.4. Determination of the Levels of cAMP, L-BAIBA, and L-Glutamate

Levels of cAMPs were monitored using UHPLC (Acquity UPLC H-Class, Waters, Milford, MA, USA) equipped with an Acquity SQ detector (Waters). In total, 5 μL aliquots (sample solution) were injected using an Acquity UHPLC Sample Manager FTN (Waters), and the analytical column was a Hypercarb (graphite carbon column, particle size = 3 μm, 150 × 2.1 mm; Thermo Fisher Scientific Inc, Waltham, MA, USA). The flow rate of the mobile phase was maintained at 450 μL/min at 40 °C. A linear gradient elution program was used for 10 min with mobile phases A (1 mM ammonium acetate buffer, pH = 11) and B (acetonitrile). Nitrogen flow for dissolution and the cone were set to 750 and 5 L/h, respectively. The temperature for dissolution was set to 450 °C. The cone voltage to detect cAMP was 42 V (*m*/*z* = 330.3).

Levels of both L-BAIBA and L-glutamate were monitored using an UHPLC (xLC3185PU, Jasco, Tokyo, Japan). Samples were automatically dual-derived using isobutyryl-L-cysteine and o-phthalaldehyde by drawing a 5 μL aliquot of the sample, standard, or blank solution and 5 μL of the derivative reagent solution and then mixed in a 386-well microplate for 5 min before injection. Derived samples (5 μL) were injected automatically using an xLC3059AS autosampler (Jasco, Tokyo, Japan). L-BAIBA and L-glutamate were separated using a Triat C18 (particle size: 1.8 µm, 50 mm × 2.1 mm; YMC, Kyoto, Japan) maintained at 35 °C. The mobile phase flow was set to 500 μL/min with a linear gradient between mobile phases A (0.1 M citrate buffer, pH = 3.5) and B (acetonitrile) for 15 min, with the excitation/emission wavelength of the fluorescence detector (xLC3120FP, Jasco) set to 345/455 nm.

### 2.5. Preparation of the Microdialysis System

To measure L-BAIBA levels in the tissues perfused in the medial prefrontal cortex (mPFC), the diffusion rate of L-BAIBA was determined with a probe according to a reverse dialysis method [17,56,57,69]. Loss of solute from perfusion medium generates at the same ratio as recovery of solute into the perfusion medium, since diffusion generates in both directions across dialysis membranes. Temperature was maintained at 37 °C using perfusate warmer. Probes were also warmed in chamber, and modified Ringer’s solution (MRS) containing L-BAIBA was infused in the probes for 3 h. The diffusion rate of L-BAIBA was approximately 10.6 ± 1.6%. Based on previous data, the systemic chronic administration of clozapine increased extracellular L-BAIBA (0.1 μM) [23]. Therefore, considering the diffusion rate of L-BAIBA, in the present study, perfusion of 1 μM L-BAIBA into the mPFC was conducted. The diffusion rate of clozapine was reported to be 9.6 ± 0.5% [18]. Therefore, to determine the therapeutically relevant concentration of clozapine, perfusion with 30 μM clozapine was performed (estimated clozapine concentration in the brain tissue was 2.9 μM).

Rats were anesthetized by 1.8% isoflurane and placed on a stereotaxic frame. Direct insertion microdialysis probe was inserted into the mPFC (A = +3.2 mm, L = +0.8 mm, V = −5.2 mm, relative to the bregma; 0.22 mm diameter, 3 mm exposed membrane; Eicom, Kyoto, Japan) and RTN (A = −1.4 mm, L = +1.2 mm, V = −7.2 mm, relative to the bregma at a lateral angle of 30°). Perfusion in probes commenced 18 h after recovery from anesthesia (constantly set at 2 μL/min) of MRS (0.145 Na^+^, 0.0027 K^+^, 0.0012 Ca^2+^, 0.001 Mg^2+^, and 0.1544 Cl^−^, buffered to pH of 7.4 with 0.002 M phosphate buffer and 0.0011 M Tris buffer). Extracellular levels of L-glutamate were measured 6 h after starting perfusion.

The perfusate in mPFC and RTN began with MRS. To determine the effects of MK801 and L-BAIBA, the perfusion medium in the mPFC was changed from MRS to MRS containing 1 μM L-BAIBA. After 1 h of perfusion of MRS containing 1 μM L-BAIBA into the mPFC, the perfusion medium in RTN was switched to MRS containing 10 μM MK801 for 120 min [57]. To determine the interaction between L-BAIBA, GABA_B_-R, and III-mGluR on MK801-evoked L-glutamate release, the perfusion medium in the mPFC was switched to MRS containing 1 μM L-BAIBA with 50 μM CGP52432 or 100 μM CPPG. After 1 h perfusion of MRS containing 1 μM L-BAIBA with 50 μM CGP52432 or 100 μM CPPG into the mPFC, the perfusion medium in RTN was switched to MRS containing 10 μM MK801. To determine the time-dependent effects of clozapine on MK801-evoked L-glutamate release, the perfusion medium in mPFC commenced with MRS containing 30 μM clozapine for 4 h or 12 h, and then the perfusion medium in the RTN was switched from MRS to MRS containing 10 μM MK801 for 120 min.

Each dialysate was injected into UHPLC to measure the levels of L-glutamate in the perfusate. Dialysis probe locations were verified after experiment by 200 μm thick brain slices (Vibratome 1000, Technical Products International INC, St. Louis, MO, USA).

### 2.6. Data Analysis

All of experiments were designed with groups (equal number of animals, n = 6), with no formal power analysis, according to our previous studies [17,18,55,57,60,70,71,72].

All values are indicated mean ± standard deviation (SD), and *p* < 0.05 was considered to be statistically significant. The agents’ level was selected according to previous reports [17,18,55,57,60,70,71,72]. Where possible, we aimed to randomize and blind the data. To determine the levels of L-BAIBA, L-glutamate, and cAMP, the sample order was selected on autosamplers using random number tables.

The time-dependent and level-dependent effects of clozapine on the intracellular concentration of L-BAIBA were analyzed via one-way analysis of variance (ANOVA) with Scheffe’s post hoc tests by BellCurve for Excel version 3.2 (Social Survey Research Information Co. Ltd., Tokyo, Japan). Level-dependent effects of L-BAIBA on accumulation of cAMP were also analyzed via ANOVA with Scheffe’s post hoc test. Effects of clozapine on MK801-evoked L-glutamate release were assessed using multivariate ANOVA (MANOVA) with Scheffe’s post hoc test (BellCurve for Excel). Interactions between L-BAIBA and inhibitors of GABA_B_-R (CGP52434) and III-mGluR (CPPG) on MK801-evoked L-glutamate release were also analyzed using MANOVA with Scheffe’s post hoc test (BellCurve for Excel). When the data did not violate the assumption of sphericity (*p* > 0.05), the F-value in MANOVA was analyzed through sphericity-assumed degrees of freedom. However, when the assumption of sphericity was violated (*p* < 0.05), the F-value was analyzed via Chi–Muller’s corrected degrees of freedom. When the F-value for any factor in MANOVA was significant, the data were analyzed using Scheffe’s post hoc test.

## 3. Results

### 3.1. Time-Dependent and Concentration-Dependent Effects of Clozapine on Intracellular L-BAIBA Levels in Astrocytes

**Figure 1:**
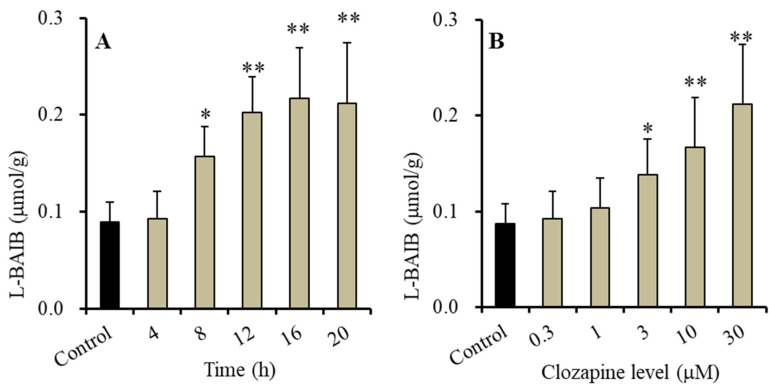
Time-dependent (**A**) and level-dependent (**B**) effects of clozapine on the intracellular levels of L-β-aminoisobutyric acid (L-BAIBA) in the astrocytes. (Panel **A**) displays the time-dependent effects of 30 μM clozapine (ranging from 4 to 20 h). (Panel **B**) indicates the level-dependent effects of clozapine (ranging from 0 to 30 μM) for 12 h. Ordinate indicates mean ± SD (n = 6) of intracellular levels of L-BAIBA (μmol/g protein). * *p* < 0.05, ** *p* < 0.01: relative to the control based on an analysis of variance (ANOVA) with Scheffe’s post hoc test.

Chronic administration (for 14 d) of therapeutically relevant concentrations of clozapine (3 μM) increased intracellular L-BAIBA levels in astrocytes [23]; however, the critical time to initiate the increase in L-BAIBA synthesis with clozapine has not been clarified. Therefore, in order to preliminarily set the temporal experimental conditions, the effects of the acute administration of clozapine on intracellular L-BAIBA levels in cultured astrocytes were determined using the maximal experimental concentration of clozapine (30 μM). The acute administration of 30 μM clozapine had time-dependent effects (F(5,35) = 27.5 (*p* < 0.01)) (Figure 1A). Overall, 4 h of exposure to 30 μM clozapine did not affect L-BAIBA levels, whereas exposure time longer than 8 h increased L-BAIBA levels. After 12 h, the increase in L-BAIBA levels reached its plateau (Figure 1A).

Based on the time-dependent features, to monitor the level-dependent effects of clozapine on the intracellular levels of L-BAIBA in astrocytes, the astrocytes were exposed to clozapine for 12 h. Acute exposure to clozapine (for 12 h) concentration-dependently increased intracellular L-BAIBA levels in the astrocytes (F(5,35) = 26.2 (*p* < 0.01)) (Figure 1B). Therapeutically relevant (3 μM) and supratherapeutic (10 and 30 μM) concentrations of clozapine increased intracellular levels of L-BAIBA, but lower-than-therapeutically relevant concentrations (0.3 or 1 μM) did not have any effects (Figure 1B). Conversely, exposure to therapeutic concentration of clozapine for 8 h did not affect intracellular L-BAIBA levels (data not shown).

### 3.2. Time-Dependent and Concentration-Dependent Effects of Clozapine on Astroglial Ripple-Evoked L-BAIBA Release

**Figure 2:**
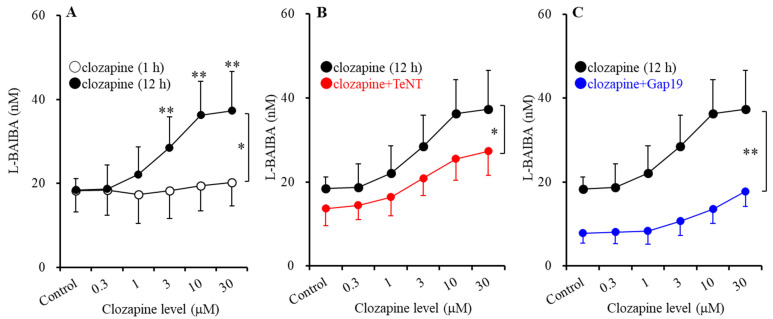
Level-dependent effects of clozapine and the interaction between clozapine and inhibitors of synaptobrevin (tetanus toxin: TeNT) and the connexin43-containing hemichannel (TAT-Gap19) on astroglial ripple-evoked L-BAIBA release. (Panel **A**) indicates the level-dependent effects of clozapine (ranging from 0 to 30 μM) for 1 h (opened circles) and 20 h (closed circles) on ripple-evoked astroglial L-BAIBA release. (Panel **B**) indicates the level-dependent effects of clozapine (0–30 μM) for 20 h (closed circles) after incubation in a cultured medium with (red circles) or without (closed circles) tetanus toxin (TeNT: 3 μg/mL) for 24 h on ripple-evoked L-BAIBA release. (Panel **C**) indicates the level-dependent effects of clozapine (0–30 μM) for 20 h (closed circles) after incubation in a cultured medium with (blue circles) or without (closed circles) Gap19 (20 μM) on ripple-evoked L-BAIBA release. Ordinate: mean ± SD (n = 6) of the extracellular L-BAIBA level (nM). * *p* < 0.05, ** *p* < 0.01: relative to clozapine exposure for 1 h based on a two-way ANOVA with Scheffe’s post hoc test.

To clarify the concentration-dependent effects of clozapine on ripple-evoked astroglial L-BAIBA release [24], astrocytes were exposed to clozapine for 1 h or 12 h. Exposure to clozapine for 12 h concentration-dependently increased astroglial ripple-evoked L-BAIBA release in a concentration-dependent manner, whereas exposure for 1 h did not have an effect (F_level_(5,50) = 41.2 (*p* < 0.01), F_time_(1,10) = 5.4 (*p* < 0.05), F_level*time_(5,50) = 28.2 (*p* < 0.01)) (Figure 2A). Notably, exposure to clozapine for 12 h ranging from 3 to 30 μM significantly increased L-BAIBA release compared to 1 h clozapine exposure. To identify the acute effects of clozapine on astroglial L-BAIBA release, such as exocytosis or non-exocytosis, we analyzed the effects of TeNT (exocytosis inhibitor via cleavage synaptobrevin) [17,60] and Gap19 (connexin43 containing hemichannel inhibitor) [19,58] on clozapine-induced L-BAIBA release. The increased astroglial L-BAIBA release induced by ripple-evoked stimulation was inhibited by both 3 μg/mL TeNT (F_level_(5,50) = 108.0 (*p* < 0.01), F_TeNT_(1,10) = 5.2 (*p* < 0.05), F_level*TeNT_(5,50) = 4.0 (*p* < 0.01)) (Figure 2B) and 20 μM Gap19 (F_level_(5,50) = 87.2 (*p* < 0.01), F_Gap19_(1,10) = 30.5 (*p* < 0.05), F_level*Gap19_(5,50) = 28.2 (*p* < 0.01)) (Figure 2C). However, astroglial ripple-evoked L-BAIBA release was dominated by non-exocytotic components (through the connexin43-containing hemichannel) over exocytotic release (Figure 2B,C).

### 3.3. Concentration-Dependent Effects of L-BAIBA on GABA_B_-R and III-mGluR

**Figure 3:**
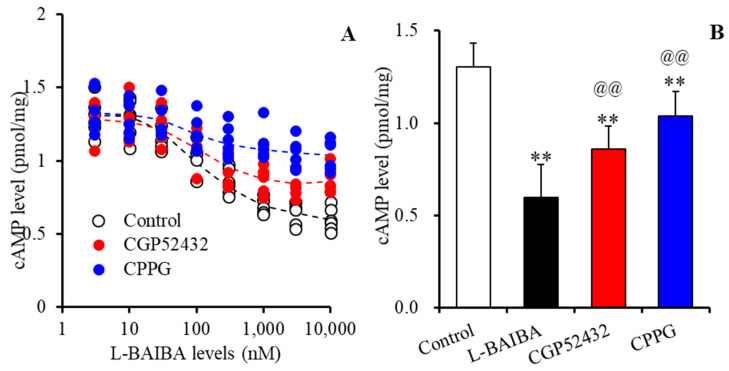
Level-dependent effects of L-BAIBA on the intracellular levels of 10 μM forskolin-evoked cyclic adenosine monophosphate (cAMP) accumulation in astrocytes, and the interaction between L-BAIBA and inhibitors of GABA_B_-R (CGP52432: 50 μM) and α-cyclopropyl-4-phosphonophenyl glycine (CPPG: 100 μM) on cAMP accumulation. (Panel **A**) indicates the level-dependent effects of L-BAIBA (ranging from 0 to 10 μM) in artificial cerebrospinal fluid (ACSF) with (red circles) or without (control: opened circles) 50 μM CGP52432 or 100 μM CPPG (blue circles). The ordinate and abscissa indicate the intracellular cAMP level (pmol/mg protein) and L-BAIBA concentration (nM), respectively. (Panel **B**) indicates the effects of 10 μM L-BAIBA, 10 μM L-BAIBA plus 50 μM CGP52432, or 10 μM L-BAIBA plus 100 μM CPPG. The ordinate indicates the intracellular cAMP level (pmol/mg protein). ** *p* < 0.01: relative to L-BAIBA alone, @@ *p* < 0.01: relative to the control, based on ANOVA with Scheffe’s post hoc test.

To clarify the concentration-dependent effects of L-BAIBA on GABA_B_-R and III-mGluR in astrocytes, the interactions between L-BAIBA and inhibitors of GABA_B_-R (50 μM CGP52434) and III-mGluR (100 μM CPPG) on 10 μM forskolin induced cAMP accumulation in astrocytes, since cAMP is the major second messenger of both GABA_B_-R and III-mGluR [61,62]. L-BAIBA decreased astroglial cAMP accumulation in a concentration-dependent manner (F(7,40) = 46.6 (*p* < 0.01)), and higher than 100 nM L-BAIBA significantly decreased cAMP accumulation (Figure 3A). Both CGP52434 and CPPG suppressed the inhibitory effects of L-BAIBA on astroglial cAMP accumulation (F_level_(7,105) = 482.8 (*p* < 0.01), F_antagonist_(2,15) = 7.8 (*p* < 0.01), F_level*antagonist_(14,105) = 32.6 (*p* < 0.01)) (Figure 3A). The inhibitory effects of L-BAIBA (10 μM) on cAMP accumulation were inhibited by 50 μM CGP52434 and 100 μM CPPG (Figure 3B). However, IC50 values of L-BAIBA weakly increased from 1.19 μM to 1.28 μM and 1.33 μM with CGP52434 and CPPG, respectively (Figure 3A).

**Figure 4:**
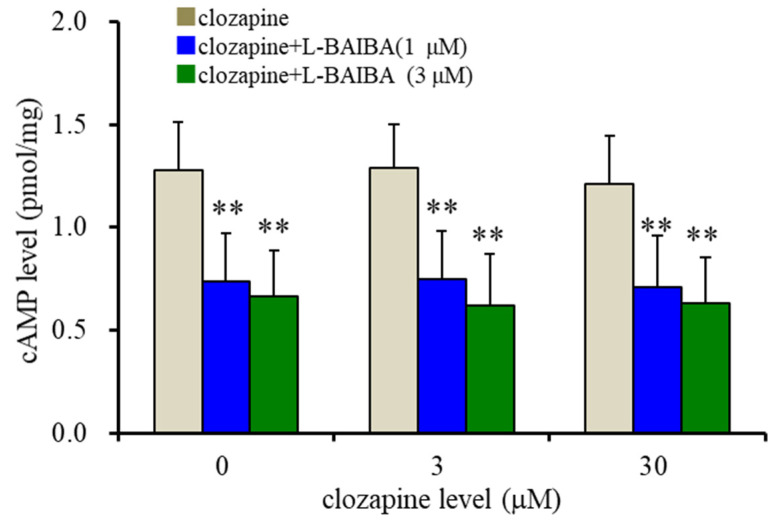
Level-dependent effects of L-BAIBA and clozapine on the intracellular levels of 10 μM forskolin-evoked cAMP accumulation in the astrocytes. The ordinate indicates the intracellular cAMP level (pmol/mg protein), and the abscissa indicates the levels of clozapine (μM). ** *p* < 0.01: relative to exposure to clozapine without L-BAIBA using two-way ANOVA with Scheffe’s post hoc test.

To determine the interaction between the concentration-dependent effects of clozapine and L-BAIBA on 10 μM forskolin-induced cAMP accumulation in astrocytes, astrocytes were exposed to therapeutically relevant (3 μM) and supratherapeutic (30 μM) concentrations of clozapine with L-BAIBA (1 and 30 μM). L-BAIBA decreased astroglial cAMP accumulation, but clozapine did not affect cAMP accumulation (F_clozapine_(2,45) = 0.2 (*p* > 0.1), F_L-BAIBA_(2,45) = 36.9 (*p* < 0.01), F_clozapine*L-BAIBA_(4,45) = 0.1 (*p* > 0.1)) (Figure 4).

### 3.4. Effects of Prompt Clozapine Discontinuation on Intracellular L-BAIBA Levels in the Astrocytes

To explore the effects of prompt clozapine discontinuation on intracellular L-BAIBA levels, after chronic exposure to therapeutically relevant (3 μM) or supratherapeutic (30 μM) dosages of clozapine, the astrocytes were incubated in fDMEM without clozapine for 3 days.

**Figure 5:**
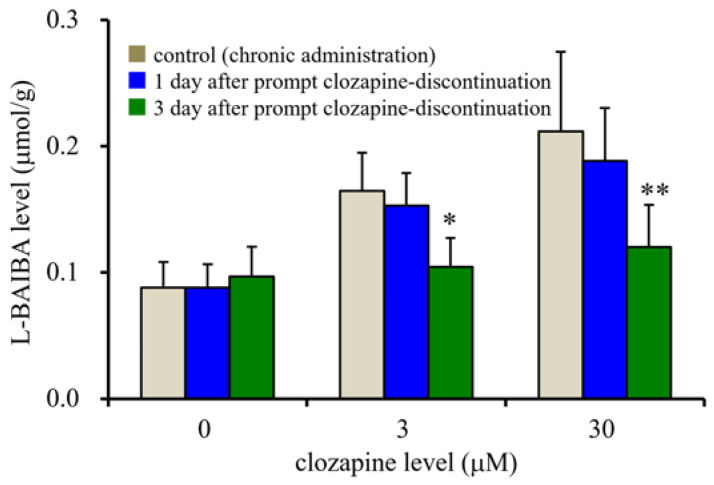
Effects of prompt clozapine discontinuation after chronic exposure to clozapine on intracellular L-BAIBA levels in the astrocytes. Ordinate: mean ± SD (n = 6) of intracellular L-BAIBA levels (μmol/g protein). * *p* < 0.05, ** *p* < 0.01, relative to the control (chronic administration before the discontinuation) based on a two-way ANOVA with Scheffe’s post hoc test.

The chronic administration of clozapine level-dependently increased intracellular L-BAIBA levels. One day after clozapine discontinuation, the increase in L-BAIBA was observed to be sustained, whereas 3 days after clozapine discontinuation, the intracellular L-BAIBA levels decreased until they almost equaled the levels of unexposed astrocytes (control) (F_clozapine_(2,45) = 27.4 (*p* < 0.01), F_discontinuation_(2,45) = 9.9 (*p* < 0.01), F_clozapine*discontinuation_ (4,45) = 3.9 (*p* < 0.01)) (Figure 5).

### 3.5. Effects of the Local Administration of Clozapine into the mPFC on MK801-Evoked L-Glutamate Release in the mPFC

The diffusion rates of clozapine into the brain tissue were detected using a reverse dialysis method to be approximately 10%, as previously reported [18]. Based on previous reports, in the present study, to study the effects of therapeutically relevant concentrations of clozapine on MK801-evoked L-glutamate release in the mPFC, 30 μM clozapine (the estimated extracellular level of clozapine was about 3 μM) was locally administered into the mPFC using the reverse-dialysis method [17,56,57,69].

**Figure 6:**
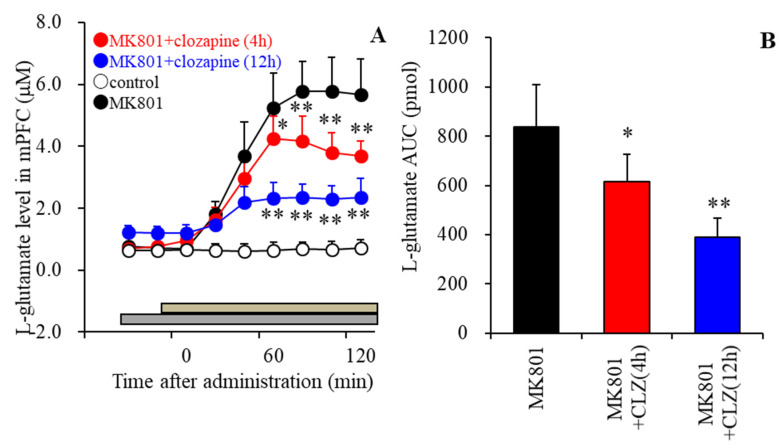
Effects of the perfusion of clozapine (CLZ) into the mPFC on L–glutamate release in the mPFC induced by the perfusion of 10 μM MK801 into RTN (MK801–evoked stimulation). Perfusion medium in the mPFC commenced with MRS with or without (control) 30 μM clozapine. After perfusion with clozapine for 4 h or 12 h (grey bars), the perfusate in the RTN was switched from MRS to MRS containing 10 μM MK801 for 120 min (brown bars). (Panel **A**): Ordinates and abscissa indicate the mean ± SD (n = 6) of extracellular L–glutamate levels (μM) and time after MK801–evoked stimulation (min). (Panel **B**) indicates the area under curve (AUC) values of MK801–evoked L–glutamate release in the mPFC (pmol). (**A**): * *p* < 0.05, ** *p* < 0.01: relative to MK801 alone based on a MANOVA with Scheffe’s post hoc test. In (**B**): * *p* < 0.05, ** *p* < 0.01: relative to the ACU of MK801 alone based on a MANOVA with Scheffe’s post hoc test.

The perfusion of 10 μM MK801 into the RTN [55,57,72] increased L-glutamate release in the mPFC (F(6,35) = 29.7 (*p* < 0.01)) (Figure 4A). The perfusion of 30 μM clozapine into the mPFC time-dependently decreased MK801-evoked L-glutamate release in the mPFC (F_time_(2.4,36.1) = 335.8 (*p* < 0.01), F_duration_(2,15) = 15.7 (*p* < 0.01), F_time*duration_(4.8,36.1) = 46.1 (*p* < 0.01)) (Figure 6A,B). The inhibitory effects of clozapine after 12 h on MK801-evoked L-glutamate release were dominant compared to those after 4 h (Figure 6B). These results suggest that the inhibitory effects of the local administration of clozapine on MK801-evoked L-glutamate release are likely mediated not only by the direct action of clozapine but also by other factors associated with the metabolic modulation induced by clozapine.

### 3.6. Effects of the Local Administration of L-BAIBA into the mPFC on MK801-Evoked L-Glutamate Release in the mPFC

**Figure 7:**
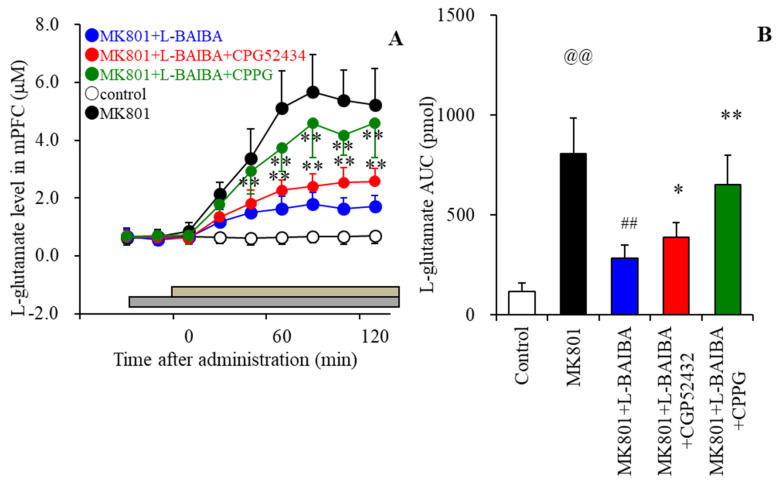
Interactions between the perfusion of 1 μM L–BAIBA and antagonists of GABA_B_–R (50 μM CGP52434) and III–mGluR (100 μM CPPG) into the mPFC on MK801–evoked L–glutamate release in the mPFC. The perfusion medium in the mPFC was based on MRS with or without (MK801) 1 μM L–BAIBA, 1 μM L–BAIBA plus 50 μM CGP52434, or 100 μM CPPG (grey bars). The perfusate in the RTN was switched from MRS to MRS containing 10 μM MK801 for 120 min (brown bars). (Panel **A**) Ordinates and abscissa indicate the mean ± SD (n = 6) of extracellular L–glutamate levels (μM) and the time after MK801–evoked stimulation (min). (Panel **B**) indicates the AUC values of MK801–evoked L–glutamate release in the mPFC (pmol). (**A**): ** *p* < 0.01: relative to MK801+L–BAIBA based on MANOVA with Scheffe’s post hoc test. (**B**): @@ *p* < 0.01 relative to the control, ## *p* < 0.01 relative to MK801, * *p* < 0.05, ** *p* < 0.01: relative to MK801+L–BAIBA by MANOVA with Scheffe’s post hoc test.

Based on the previous pre-clinical findings that clozapine enhances the function of GABA_B_-R and III-mGluR [18,73], to clarify the effects of L-BAIBA on these receptors, we determined the interactions between L-BAIBA and the antagonists of GABAB-R on GABA_B_-R and III-mGluR on MK801-evoked L-glutamate release. The perfusion of 1 μM L-BAIBA into the mPFC decreased MK801-evoked L-glutamate release in the mPFC (F_time_(2.7,27.3) = 73.4 (*p* < 0.01), F_BAIBA_(1,10) = 77.6 (*p* < 0.01), F_time*BAIBA_(2.7,27.3) = 70.9 (*p* < 0.01)) (Figure 7A,B). Perfusion of 50 μM CGP52434 (GABA_B_-R antagonist) into the mPFC suppressed the inhibitory effects of L-BAIBA on MK801-evoked L-glutamate release in the mPFC (F_time_(4.9,48.8) = 192.6 (*p* < 0.01), F_CGP_(1,10) = 5.4 (*p* < 0.05), F_time*CGP_(4.9,18.8) = 18.6 (*p* < 0.01)) (Figure 7A,B). The perfusion of 100 μM CPPG (III-mGluR antagonist) into the mPFC also suppressed the inhibitory effects of L-BAIBA on MK801-evoked L-glutamate release in the mPFC (F_time_(2.1,20.7) = 113.7 (*p* < 0.01), F_CPPG_(1,10) = 28.3 (*p* < 0.01), F_time*CPPG_(2.1,20.7) = 39.4 (*p* < 0.01)) (Figure 7A,B).

## 4. Discussion

This study demonstrated that the sub-acutely persistent exposure of clozapine to primary cultured cortical astrocytes concentration-dependently increased L-BAIBA synthesis. The increased L-BAIBA reached a steady state after 12 h. We observed an increase in intracellular L-BAIBA, which is released as a gliotransmitter through exocytosis and activated hemichannels and regulates tripartite synaptic transmission as an endogenous agonist of GABA_B_-R and III-mGluR. These results explain, at least partially, the mechanism behind the stimulatory effects of clozapine on GABA_B_-R and III-mGluR, which has been implicated as a potential mechanism underlying the effects of clozapine in treatment-resistant schizophrenia [18,50,51]. The impacts of L-BAIBA on the pharmacological action of clozapine are discussed below.

Our recent study already reported that the chronic administration of therapeutically relevant doses of clozapine increased L-BAIBA in the hypothalamus [23]. Increased intracellular L-BAIBA in the hypothalamus contributes to type A adverse effects to clozapine, such as weight increase and metabolic disturbances, by increasing hypothalamic AMPK signaling [23,37]. However, the present study suggests that increases in L-BAIBA synthesis caused by clozapine are possibly involved not only in adverse effects but also in clozapine’s mechanisms of efficacy in treatment-resistant schizophrenia through the agonistic action of L-BAIBA against GABA_B_-R and III-mGluR. Therefore, the increased signaling in both intracellular and extracellular L-BAIBA likely plays important roles in the mechanisms of clinical action of clozapine as a double-edged sword. The BAIBA enantiomers L-BAIBA and D-BAIBA are GABA isomers, but their synthesis pathways are rather different. L-BAIBA and D-BAIBA are synthesized from L-valine and thymine, respectively [74,75,76,77]. Indeed, in the rat body, D-BAIBA was found to be the dominant BAIBA enantiomer in the plasma, whereas in the CNS, L-BAIBA was the dominant enantiomer, but levels of D-BAIBA could not be detected [23,24]. Based on previous findings, in the present study, functional analysis was conducted only on L-BAIBA.

Clozapine is the sole approved antipsychotic agent for the treatment of antipsychotic-resistant schizophrenia and remains one of the most effective antipsychotic agents [1,2,3,4,5]. Clozapine was developed and approved for the treatment of schizophrenia in the 1970s [78]. Although the pathophysiology of schizophrenia has developed over the last fifty years, the detailed mechanism underlying the efficacy of clozapine in treatment-resistant schizophrenia remains to be clarified [78]. In other words, the specific mechanisms of clozapine that are existent in other atypical antipsychotics have not been elucidated [78]. A pharmacological schizophrenia model induced by non-competitive NMDA-R antagonists, such as phencyclidine, ketamine, and MK-801, exhibit certain features of schizophrenia, such as negative symptoms and cognitive deficits, more closely than those in amphetamine/dopamine-induced psychosis models [11]. The non-competitive NMDA R antagonists, phencyclidine and ketamine, also clinically generate schizophrenia-like positive/negative symptoms in healthy individuals and exacerbate psychosis in patients with schizophrenia [79].

The systemic administration of MK801 drastically activates thalamic glutamatergic neuronal activities by suppressing intra-thalamic GABAergic transmission (from RTN to the mediodorsal thalamic nucleus: MDTN), resulting in increased L-glutamate release in several frontal regions, including the mPFC and orbitofrontal cortex [72,80]. It was established that the hyperactivation of glutamatergic transmission in thalamocortical pathways from MDTN to several frontal cortex regions plays important roles in the dysfunction of several cognitive components such as sensory integration and executive function, according to the hypothesis of bottom-up cognition promoting systems [81]. Indeed, tonic activation of thalamocortical glutamatergic transmission has been observed in several experimental animal models, such as schizophrenia, attention-deficit hyperactivity disorder, and autism [18,56,70,82,83,84]. The local administration of several antipsychotics into MDTN was already demonstrated to inhibit MK801-induced tonic activation of glutamatergic transmission in the thalamocortical pathway by suppressing glutamatergic neuronal activity in the MDTN [55,57,85]. Conversely, local administration of clozapine into the mPFC suppressed MK801-induced hyper-glutamatergic transmission [18]. Thus, the mechanisms related to the suppressive effects of clozapine on hyper-activated thalamocortical glutamatergic transmission are notably different than those of other antipsychotics, with other antipsychotics targeting the thalamus and clozapine targeting the frontal regions [18]. The activation of GABA_B_-R and III-mGluR was considered to be a candidate mechanism underlying clozapine’s effectiveness in treatment-resistant schizophrenia [18,50]. Both GABA_B_-R and III-mGluR are expressed in the mPFC, and the activation of these receptors suppresses transmitter release in the mPFC [86,87]. Considering these previous findings, the present results reveal that the activation of both GABA_B_-R and III-mGluR in the mPFC plays important roles in the suppressive effects of clozapine on MK801-induced hyper-activated thalamocortical glutamatergic transmission. Therefore, the activation of both GABA_B_-R and III-mGluR in the mPFC is at least partially involved in clozapine’s effectiveness in treating antipsychotic-resistant schizophrenia through the compensation of hyper-activated thalamocortical glutamatergic transmission. However, similar to previous findings [52,53,54], the present study also suggests that clozapine does not directly bind to III-mGluR or GABA_B_-R. However, exposure to clozapine for several hours suppressed MK801-evoked glutamate release in the mPFC via the activation of III-mGluR and GABA_B_-R. These inhibitory and stimulatory effects of clozapine on thalamocortical glutamatergic transmission and L-BAIBA synthesis, respectively, were found to be temporally and negatively correlated. These temporal similarities in the effects of clozapine between L-BAIBA synthesis and the inhibition of MK801-evoked glutamate release via the activation of III-mGluR and GABA_B_-R indicate that the increased astroglial transmission associated with L-BAIBA induced by clozapine possibly contributes to the improvement and/or compensation of cognitive impairments induced by the tonic activation of thalamocortical hyper-glutamatergic transmission.

According to the FDA, the recommended average cumulative daily dose of clozapine is 300~400 mg (with 2~3 doses per day, according to its short plasma half-life of 12~16 h) [88]. Considering this evidence, in clinical settings, enhanced L-BAIBA signaling in the steady-state of patients chronically taking oral clozapine may contribute to the clinical efficacy of clozapine. Conversely, a portion of the pathophysiology of clozapine-discontinuation syndrome could also be explained by the effects of clozapine on L-BAIBA. Prompt clozapine discontinuation due to several clinical scenarios, i.e., severe type B reactions or voluntary discontinuation by patients, can precipitate the sudden emergence of psychotic symptoms, which have been termed “clozapine-discontinuation syndrome” [9,10,89,90]. Clozapine-discontinuation catatonia is considered to be one of the worst prognoses in catatonic syndrome, due to the high prevalence of comorbid of rhabdomyolysis and benzodiazepine resistance [10,89,90,91]. These distinct symptomatic features of clozapine-discontinuation catatonia, such as benzodiazepine resistance, suggest that the pathophysiology of clozapine-discontinuation catatonia is involved in different mechanisms compared to other catatonic syndromes, such as idiopathic lethal catatonia, benzodiazepine-withdrawal catatonia, and neuroleptic malignant syndrome, possibly induced by the dysfunction of GABA_B_-R [9,10,51,91,92]. Therefore, the present study may also indicate a candidate mechanism for clozapine-discontinuation catatonia induced by GABA_B_-R hypofunction upon prompt discontinuation of clozapine, despite clozapine not directly binding to GABA_B_-R.

In the present study, to explore the pathophysiology of clozapine-discontinuation catatonia, the effects of the prompt discontinuation of clozapine’s exposure to astrocytes on intracellular L-BAIBA levels were also determined. Increased intracellular L-BAIBA levels in the astrocytes persisted for approximately 3 days after the prompt discontinuation of clozapine. The fact that the continued activation of L-BAIBA synthesis lasted only several days after prompt discontinuation followed by chronic exposure to clozapine indicates that the stimulatory effects of clozapine on L-BAIBA synthesis are reversible and may be involved in the pathophysiology of clozapine-discontinuation syndromes and catatonia via withdrawal from not only GABAB-R activation but also III-mGluR activation [9,10,18,50,51,92]. The chronic exposure of clozapine to cultured astrocytes followed by prompt clozapine discontinuation, as explored in this study, may not reflect the actual pharmacokinetic and/or pharmacodynamic processes underling clinical scenarios of clozapine-discontinuation catatonia. In other words, the clinical settings of clozapine-discontinuation catatonia/syndromes feature more gradual withdrawal compared to those observed under the present study design due to its use of a pharmacokinetically steady state of clozapine. Therefore, to clarify the detailed pathophysiology of clozapine-discontinuation catatonia, future in vivo studies should be conducted to validate several symptoms and identify further signaling abnormalities induced by prompt clozapine discontinuation following the chronic administration of clozapine.

## 5. Conclusions

The present study revealed that a candidate gliotransmitter and GABA isomer, L-BAIBA, activates with GABA_B_-R and III-mGluR as its agonists, but clozapine does not bind to these receptors. However, clozapine increases L-BAIBA synthesis in the astrocytes. Therefore, clozapine indirectly activates both GABA_B_-R and III-mGluR through the activation of astroglial transmission associated with L-BAIBA. The local administration of L-BAIBA into the mPFC inhibited MK801-evoked L-glutamate release through the activation of GABA_B_-R and III-mGluR. Clozapine also time-dependently suppressed MK801-evoked L-glutamate release in the mPFC through the activation of GABA_B_-R and III-mGluR. The time dependence was synchronized with an increase in L-BAIBA synthesis in the astrocytes. Therefore, increasing astroglial L-BAIBA transmission plays important roles in the pathophysiology of clozapine in terms of its effectiveness in treating antipsychotic-resistant schizophrenia.

## Data Availability

The data that support the findings of this study are available from the corresponding author upon reasonable request. Some data may not be made available due to ethical restrictions.
